# Impact of Social Support on the Functioning of Patients Receiving Home Nursing Care

**DOI:** 10.3390/ijerph22071060

**Published:** 2025-07-02

**Authors:** Bożena Ewa Kopcych, Paweł Falkowski, Daniela Patricia Santos Costa

**Affiliations:** 1 Faculty of Health Sciences, University of Lomza, 18-400 Lomza, Poland; 2 Public Palliative Care Facility in Suwałki, Home Mechanical Ventilation Team, 16-400 Suwałki, Poland; 3 Department of Family Medicine, Medical University of Bialystok, 15-089 Bialystok, Poland; pawelfalkowsky@gmail.com; 4 Polish Dermatoscopy Group, 60-355 Poznan, Poland; 5 Home Health Care, Ministry of National Guard-Health Affairs, Riyadh 11426, Saudi Arabia; dany.costa_17@hotmail.com

**Keywords:** patient, home care, social support

## Abstract

The type of non-professional or professional support received affects the quality of life of the patient and their caregivers. Social support is the type of interaction that is taken by the patient and his caregivers in a problematic, difficult, stressful, or critical situation. Aim: The aim of the study was to assess the impact of social support on the functioning of patients under nursing home care. Material and methods: The study included 148 chronically ill patients under home nursing care. The study used the diagnostic survey method; the research technique was a questionnaire containing basic data about the respondent and the Social Support Scale (SWS) by Krystyna Kmiecik-Baran. Results: The need to continue the causal treatment at home means that the main source of support for care beneficiaries are nurses who provide medical services at the patient’s home, supported by doctors and family members of the patient. According to patients’ subjective assessment of the support they received from nurses, patients rated the informational support provided by nurses highest at 14.3 points and emotional support at 13.3 points (SD 1.776). on a scale where the maximum score was 16 points. In the opinion of the surveyed patients, the value-added support provided was the lowest-rated category by patients, 9.74 points (SD 2.505). Instrumental support was also rated very poorly by the respondents (10.17 points (SD 2.069). In each category, there was no statistically significant difference at the *p* < 0.05 level in the respondents’ evaluation, which means that the expressed opinion on each type of support from the highest to the lowest evaluation: informational, emotional, instrumental, and evaluative—overlapped in the patient group and the family group. Conclusions: Patients under home care highly appreciated the support provided to them by the nursing staff. Social support for a chronically ill person who requires constant care and care by the nursing staff is a form of direct impact that relieves stress and tension, minimizes the effects of the disease, directly affects the course of treatment and care, and prevents stigmatization.

## 1. Introduction

Modern times are characterized by dynamics in many branches of popular, social, and health care life, the image of which is depicted in the words of Głowacki “widespread dissatisfaction and disillusionment have become common, European and worldwide”. The health care system is the one for which there are the highest expectations. It is defined as “an organized and coordinated set of activities, the purpose of which is the implementation of preventive and therapeutic nursing and rehabilitative services, determining and improving the health of the individual, and, in the end, the whole society [1]. The extension of human lifespan, medical advances, and the financial aspect have contributed to the development of the sector of nursing services provided in the home environment. One of the main elements of health services taking place in direct contact with the patient is nursing care. The quality of services provided in health care translates not only into the provision of functional well-being but, most importantly, into responsibility for the life and safety of the patient. Social support towards patients and their families/caregivers plays an important role in the adaptation process to changed not only health but also social conditions [2,3].

With the onset of chronic disease and its limitations, both the patient and his caregivers experience a change in the functioning of the home environment in every facet of life. These changes mainly affect the somatic, emotional, spiritual, and social spheres. They influence the search for effective solutions and behaviors aimed at maintaining a good quality of life for both care recipients and their implementers in the immediate environment [3,4]. The type of non-professional or professional support received influences the quality of life of the patient and his caregivers. Social support is a type of interaction that is taken by the patient and his caregivers in a problematic, difficult, stressful, or critical situation. The purpose of support in nursing home care is to help solve the problem, keep the patient safe, and overcome the difficult situation. Depending on the assessment of the needs of the patient’s environment, the following types of social support are distinguished:

✓ informational; consisting of providing the patient and his caregivers with important news, advice, counselling, etc. for their functioning,
✓ instrumental; consisting of providing the individual with concrete assistance, such as lending money, shopping,
✓ appreciative; involving letting the individual know that he or she has such capabilities (abilities, skills, etc.) that are important for the proper functioning of the environment or person,
✓ emotional; consisting of letting the individual know that he or she can always count on this particular group or person and will be helped at any time. Provided by doctors, nurses, and family [5,6,7,8].

According to the WHO [9] definition, social support is the help and support that individuals receive from other members of society, including family, friends, neighbors, and professional groups. It is an important factor in mental health and well-being, and a lack of adequate support can negatively affect quality of life.

### Aim

The purpose of the study was to measure and evaluate the social support received by patients and their families/caregivers receiving nursing home care.

## 2. Material and Methods

The study was conducted in the home environment of patients under home nursing care and their caregivers. The interviewers, for the purposes of this study, were individuals who did not perform nursing functions for the home communities surveyed. Prior to the start of the study, the interviewers were trained in the research topic and the procedures involved in completing the survey questionnaire. The primary inclusion criterion was the informed consent of the patient under home nursing care and his/her caregiver to participate in the study; the exclusion criterion was the patient’s inability to provide an independent response related to his/her clinical condition (coma, vegetative state), mental illness, or incompletely completed survey questionnaire. Respondents were informed that participation in the study was voluntary and anonymous. They were given additional information on the rules for completing the survey questionnaire. The study was carried out using a standardized questionnaire of the Polish adaptation of the Multidimensional Scale of Perceived Social Support developed by K.Kmiecik-Baran on four types of social support: informational, instrumental, valuing, and emotional—provided by doctors, nurses, and family, as well as a questionnaire of the authors own allowing the collection of socio-demographic data. This scale allows us to determine the outcome of social support by individual persons implementing patient care. In the study, 16 items comprising the final version of the scale were analyzed. The following rating scale was adopted for the analysis: 4—yes—a statement very strongly saturated with a given type of support, 3—rather yes—a statement strongly saturated with a given type of support, 2—rather no—a statement weakly saturated with a given type of support, 1—no, not applicable—a statement not at all saturated with a given type of support. In the case of positive statements, the respondent receives 4 points for each “yes” answer, for “rather yes” 3 points, for “rather not” 2 points, for “no” and “not applicable” 1 point. For negative statements, the scoring is reversed. The Social Support Scale allows the respondent to obtain five scores (for each support group). An overall score to determine the level of social support without differentiating it into different types of support. The total score on the scale ranges from 16 to 64 points. Higher scores indicate a better perception of social support. For the purposes of interpretation, the following cut-off points were used: scores up to 32 points were classified as indicating a very low level of social support, scores between 33 and 47 points as a medium level, and scores from 48 to 64 points as a high level of social support.

1. Score indicating the level of information support—a maximum of 16 points could be obtained, indicating a very high level of information support; a minimum of 4 points could be obtained, indicating no information support;

✓ 4 to 7 points will indicate a low level of information support,
✓ 8 to 12 points medium level of information support,
✓ 13 to 16 points high level of information support.

2. Score indicating the level of instrumental support—the maximum score could be 16 points, which indicates a very high level of information support; the minimum score could be 4 points, which indicates no instrumental support;

✓ 4 to 7 points indicate a low level of instrumental support,
✓ 8 to 12 points medium level of instrumental support,
✓ 13 to 16 points high level of instrumental support.

3. Score indicating the level of appreciative support—a maximum of 16 points could be obtained, which indicates a very high level of appreciative support; a minimum of 4 points could be obtained, which indicates a lack of appreciative support;

✓ 4 to 7 points indicate a low level of value support,
✓ 8 to 12 points medium level of value support,
✓ 13 to 16 points a high level of appreciative support.

4. Score indicating the level of emotional support—a maximum of 16 points could be obtained, indicating a very high level of value support; a minimum of 4 points could be obtained, indicating no emotional support;

✓ 4 to 7 points will indicate a low level of emotional support,
✓ 8 to 12 points medium level of emotional support,
✓ 13 to 16 points high level of emotional support.

Social support was assessed by relating them to the Sten norms. The following categorization was used to evaluate the scores:

✓ Sten 1–3 low scores,
✓ Sten 4–7 average scores,
✓ Sten 8–10 high scores.

Cronbach’s alpha value reported in publications is >0.7 [10,11].

Data obtained from the questionnaire were entered into an Excel spreadsheet. Statistical analysis of the data was performed using STATISTICA v.12.0 PL. The study was conducted in accordance with the Declaration of Helsinki, and the protocol was approved by the Bioethics Committee of the Medical University of Bialystok Faculty of Health Sciences R-I-002/238/2014.

## 3. Results

332 correctly completed survey questionnaires were used to present the results in measuring and evaluating the social support received toward patients and their families/caregivers receiving nursing home care. Two groups of respondents were surveyed:

Group I—Patient (148 questionnaires) is a chronically ill patient residing in a home setting towards whom home care nurse services were implemented. These included mechanically ventilated patients with neuromuscular diseases in an invasive form (8 subjects) or by a non-invasive method (20 patients); 74 subjects receiving long-term care as a result of disease complications requiring the provision of systematic medical services (hard-to-heal wounds, Foley catheter, stomas); 16 subjects receiving palliative care as a result of the end of the treatment process; and 30 subjects receiving systematic services from a family nurse practitioner.

Group II—Family (184 questionnaires) are family members of patients or their caregivers who perform caregiving functions.

### 3.1. Sociodemographic Assessment Results

The statistical calculations presented in the tables used a comparison of the distribution of responses between the responses of the patient and family groups. A *p*-value ≤ 0.05 in Pearson’s chi-square test was used for statistical calculations and analyses.

The study included 148 chronically ill patients receiving home nursing services. The study group was predominantly female 75% of the time. The Family group had 184 respondents; 82.6% of the caregivers were women. Men accounted for only 20% of the respondents. The distribution of data are shown in Table 1.

The respondents in the study groups had the highest number of patients aged over 70 years (33.8%) and 61–70 years (19.6%) and caregivers aged 51–60 years (36.4%) and 41–50 years (20.1%). The youngest patient was under 20 years of age; caregivers in the <20 years range accounted for 1.6% of the subjects. The crosstabulation table shows statistically significant differences (*p* = 0.00) of the variables for the Patient and Family group with respect to age structure. The main place of residence in both the Patient group (85.8%) and the Family group (83.2%) of respondents was the city. The variables included in the questionnaire regarding the residence structure of chronically ill patients included the following modes of residence: alone, with wife/husband only, with wife/husband with children, with children only, alone, and other. The data showed that solitude was predominant among patients (36.1%). Shared household with children was shown by 12.8% of respondents. The Family group was characterized by the structure of both spouses in 48.4%, with respect to the sick person’s children, 11.4%. Other people living with the respondents were 9.5% of the respondents. In the respondents’ self-assessment, average social and living conditions occurred in 45.5% and good in 38.0% of respondents. Poor material situation concerned 13.5% of the total respondents. The situation was very bad, as indicated by 3.0% of the total respondents. There were no statistically significant differences in material situation between responses in the surveyed groups. The nurse in 67.5% most often implemented medical care, according to respondents of both study groups, 62.2% in the Patient group and 71.7% in the Family group; the differences were not statistically significant. Respondents were followed by a doctor (54.8%), with the lowest percentage of responses received by a psychologist (1.8%). The family provided care to its members in only 15.1%. Table 2.

### 3.2. Social Support

#### 3.2.1. Social Support for the Home Environment Provided by the Doctor

Based on the analysis of the averages, we can conclude that the least support from the medical staff was given to patients and their families/caregivers in terms of value support (8.99 vs. 9.22 points) and instrumental support (9.49 vs. 9.39 points), while the most was given to informational (13.45 vs. 13.31 points) and emotional support (12.88 vs. 12.68 points). Valuing support, according to patients, refers to a type of social support that aims to improve the supported person’s self-esteem and self-concept. The lack of emphasis on the patient’s strengths and achievements affects the perception of his positive qualities and skills. This also applies to family/caregiver respondents. Reassuring and mobilizing the patient and family that they are able to meet challenges and cope with difficulties increases their confidence. Instrumental support, whose role is to help, with the goal of providing specific resources and skills so that they can better cope with daily functioning and nursing and treatment activities. This area needs to be made more effective. As can be seen from the data obtained, we can see that the evaluation of the support provided in each category overlapped in the assessment of patients and their caregivers. Summarizing the obtained data, we can conclude that in the opinion of the surveyed patients and their family caregivers, the social support provided by the doctor did not differ significantly in each of the analyzed categories Table 3.

#### 3.2.2. Social Support Provided by Home Care Nurses

The data clearly indicate that, in the opinion of the patients surveyed, the value support given regarding showing appreciation and acceptance of the importance of the home care nursing patient was the lowest-rated category by patients and families (9.74 vs. 10.33 points). Instrumental support, as the overriding element in nursing home care, whose function is to show with instruction and teach self care, was also rated very low by respondents of both groups (10.17 vs. 10.55 points). The highest rating was given to informational support, whose task was to provide advice and guidance, share experiences to enable patients and their caregivers to deal appropriately with illness and disability in order to accept chronic disease and the resulting handicaps, and inform them about institutions and organizations that support home environments in difficult situations. Emotional support was also rated highly by respondents (13.3 vs. 13.45 points). In each category, there was no statistically significant difference at the *p* < 0.05 level in the respondents’ evaluation, which means that the expressed opinion on each type of support from the highest to the lowest rating: informational, emotional, instrumental, and valuational overlapped in the Patient group and the Family group. Both patients and their families rated informational and emotional support the highest, while valuing instrumental support the lowest. At the same time, the lack of statistical significance of the differences between the ratings of the two groups allows us to conclude that perceptions of the quality of each type of support were consistent, which may indicate consistent experiences and expectations of nursing home care. This is very important in terms of service planning—there is no need to differentiate support according to the recipient (patient vs. caregiver). Detailed data are shown in Table 4.

#### 3.2.3. Social Support Provided by the Family

Based on the results of the analysis, it was found that there was a statistically significant difference in the level of informational support provided by family members/caregivers and the Patient group *p* < 0.05; r = −0.242. The mean level of support provided in the Family group was lower than the informational support provided in the Patient group (M = 12.71, SD = 2.729 vs. M = 13.33, SD = 2.803). The differences between these measurements were small, as found by the result of r = −0.242. In the other categories, there was no statistically significant difference at the *p* < 0.05 level. The lowest rating was given to value support in both study groups at the level of (Family 10.67 vs. Patient 10.39), which we can consider as an effect of contemporary changes taking place in the family structure. The exact distribution of responses is shown in Table 5.

## 4. Discussion

Numerous scientific publications [10,11,12] indicate that chronic illness and lack of self-care surprise everyone regardless of age, gender, or location. It involves shock, severe stress, and loss of a sense of security and independence. Losing control over one’s own body, but also over one’s own life and the world around, is a traumatic experience for many people. It depresses and frightens them, so patients become apathetic and unpleasant to loved ones and limit contact with other people. The availability of social support helps in recovery. On the other hand, those who lacked social support felt that this contributed to difficulties in recovery. This included difficulties in obtaining and understanding medical instructions [13,14,15].

Services provided in the home setting are carried out by nurses in long-term home care teams for adults, children, and adolescents for patients who have scored 0 to 40 points on the Barthel scale and do not qualify for inpatient treatment but require systematic and intensified nursing care. The services provided by long-term nursing care include nursing, rehabilitation, and assistance in solving biological, psychological, and social problems.

Social support, as reported in numerous research reports [16,17,18,19], both in achieving health and optimal quality of life and in coping with illness, occupies a high position. It is a multidimensional construct. It plays an important role in the process of coping with stressful situations. Social support in a situation of illness or disability is understood as a specific way and type of helping sick people and their families to mobilize their own strength and resources so that they themselves want and can cope with their own problems. The primary source of support for the sick person is his family and those associated with him (friends, acquaintances, neighbors) who maintain emotional contact with him. Secondary sources of support are state institutions and self-help associations. In a situation of intractable and chronic illness, social support takes on particular importance. The type and form of support provided are determined by the dynamics of the disease and the well-being and needs of the patient and his caregivers. Support flowing from family and loved ones facilitates the ability to cope with stressful and sometimes impossible life situations. However, for many people, supportive relationships are not available when they are needed most [17]. The support provided by nurses plays an important role in coping with more than just health problems, provided that the help offered is expected and relevant. With regard to our own research, respondents in both groups indicated the information support received from the nurse as the most important (Patient—14.03; Family—13.78). The result obtained indicates that the information support expected by the Patient group from the home care nurse was fulfilled in 87.69%. The Family group reported that their expectations were met 86.12%. The lowest level of support was provided by home care nurses in value support (Patient—60.87%, Family—64.56%). The result obtained informs the home care nursing community of the need to look for new solutions and behaviors to help shape both patients and their caregivers’ self-esteem, adequate strength, and belief in their own ability to cope with illness and disability, which will significantly contribute to finding internal personal resources to facilitate coping with difficult situations. Following Kowalczyk-Fobka [16], the literature emphasizes that the emotional and appreciative support given to both the patient and his caregivers should be the most important one. According to Kózka [18], this support means showing understanding and compassion for the patients autonomy. The results of a study conducted by Izdebski et al. [19] prove that the support provided by doctors and nurses in terms of information and instruments is of particular importance for the partners of women with cancer. The overall assessment of the social support provided by nurses, according to the respondents, was the fulfillment of expectations in 78.73% in the Patient group and 76.36% in the Family group, which is confirmed by the study of Lorencowicz and associates [20,21,22,23].

In the aspect of the expectations of patients and their caregivers in relation to doctors and other members performing care functions for the family member, they can count on the support of doctors in 73.44% of patients and 71.93% of caregivers, and the family provides support for the patient in 75.91% and for itself in 74.59%.

In the context of health care, appreciative support is important in building relationships with patients and improving their well-being and motivation to perform. Doctors and medical staff can use appreciative support techniques to help patients cope with their illness, improve their self-esteem, and increase their chances of recovery. The results of the survey indicate that cognitive and emotional support receives the greatest recognition in nursing home care, while practical and value-based support needs improvement. The concordance of ratings between patients and families shows that both recipients and their relatives have similar expectations and experiences, which may facilitate the implementation of uniform standards of care.

Summarizing the analyses made, it can be concluded that respondents receive social support at the level of Patient—76.02%, Family—74.29% in all types of support. The results of the survey do not confirm the existence of differences in terms of seeking or needing support between the surveyed groups. According to the surveyed patients, nurses provide social support in the categories from the highest indicated to the lowest; informational, emotional, instrumental, and valuing. The family/caregiver group showed a slight difference, indicating emotional support in second place.

## 5. Conclusions

1. The role of modern nursing in the health care sector is undergoing permanent change and evaluation.
2. The implementation of guaranteed services by a home care nurse to a chronically ill patient is aimed at preparing the patient and his caregivers for care and nursing, including the formation of skills for coping with self-care deficits.
3. The support received from nurses plays a key role in the healing process, as it increases the level of knowledge and skills in coping with the limitations of the disease, as well as influences the formation of attitudes aimed at accepting the disease and its limitations.
4. Patients expect a higher level of social support from nursing staff in each category.
5. Nurses, by providing targeted, expectant support, can positively influence their acceptance of the disease and improve their quality of life.

## Figures and Tables

**Table 1:** Gender of respondents.

			Group		Total	*p*-Value
			Patient	Caregiver		
Sex	Men’s	*n*	37	32	64	0.089
		%	25.0%	17.4%	20.8%	
	Women	*n*	111	152	263	
		%	75.0%	82.6%	79.2%	
Total		*n*	148	184	332	
		%	100.0%	100.0%	100.0%	

*p* < 0.05 in the test chi2 Pearsona.

**Table 2:** Providers of medical care for chronically ill patients.

Variables Analyzed		Group		Total	*p*-Value
		Patient	Caregiver		
Doctor	*n*	102	80	182	0.000
	%	68.9%	43.5%	54.8%	
Nurse	*n*	92	132	224	0.064
	%	62.2%	71.7%	67.5%	
Psychologist	*n*	1	5	6	0.165
	%	0.7%	2.7%	1.8%	
Rehabilitator	*n*	15	33	48	0.045
	%	10.1%	17.9%	14.5%	
Family	*n*	16	34	50	0.052
	%	10.8%	18.5%	15.1%	
Volunteer	*n*	1	1	2	0.877
	%	0.7%	0.5%	0.6%	
Other	*n*	0	2	2	0.203
	%	0.0%	1.1%	0.6%	

*p* < 0.05 in the test chi2 Pearsona.

**Table 3:** Social support provided by the doctor to patients and their caregivers.

Social Support Scale Doctor		*n*	Average	SD	Min	Mediana	Max	*p*-Value
support information	patient	148	13.45	1.942	7	14.00	16	0.606
	caregiver	179	13.31	2.078	6	13.00	16	
support instrumental	patient	148	9.49	2.275	4	9.50	16	0.732
	caregiver	179	9.39	2.126	4	9.00	16	
support valuing	patient	148	8.99	2.531	4	9.00	16	0.361
	caregiver	179	9.22	2.855	4	9.00	16	
support emotional	patient	148	12.88	1.930	8	13.00	16	0.625
	caregiver	179	12.68	2.285	6	13.00	16	
Suport total	patient	148	44.80	5.964	25	45.00	61	0.905
	caregiver	179	44.60	7.095	24	45.00	62	

*p* < 0.05 in the test Manna-Whitneya.

**Table 4:** Assessment of social support provided by home care nurses.

Social Support Scale Nurse		*n*	Average	SD	Min	Mediana	Max	*p*-Value
support information	patient	148	14.03	1.776	7	14.00	16	0.394
	caregiver	181	13.78	2.050	4	14.00	16	
support instrumental	patient	148	10.17	2.065	4	10.00	16	0.119
	caregiver	181	10.55	2.069	4	11.00	16	
support valuing	patient	148	9.74	2.505	4	10.00	16	0.061
	caregiver	181	10.33	2.666	4	10.00	16	
support emotional	patient	148	13.30	2.206	7	13.00	16	0.475
	caregiver	181	13.45	2.262	4	13.00	16	
Suport total	patient	148	47.24	5.740	25	47.00	60	0.096
	caregiver	181	48.11	6.878	19	49.00	63	

*p* < 0.05 in the test Manna-Whitneya.

**Table 5:** Assessment of social support provided by the caregiver.

Social Support Scale Caregiver		*n*	Average	SD	Min	Mediana	Max	*p*-Value
support information	patient	148	13.33	2.803	4	14.00	17	0.015
	caregiver	182	12.71	2.729	4	13.00	16	
support instrumental	patient	148	11.98	2.970	4	12.00	16	0.059
	caregiver	182	11.29	3.048	4	12.00	16	
support valuing	patient	148	10.39	3.471	4	10.00	16	0.542
	caregiver	182	10.67	3.202	4	11.00	16	
support emotional	patient	148	12.88	3.160	4	13.00	16	0.955
	caregiver	182	13.07	2.763	4	13.00	16	
Suport total	patient	148	48.58	10.131	19	50.00	64	0.293
	caregiver	182	47.74	9.548	22	49.00	64	

*p* < 0.05 in the test Manna-Whitneya.

## Data Availability

Derived data supporting the findings of this study are available from the corresponding author upon request.

## References

[1] Krukiel A., Sienkiewicz Z., Wrońska I. (2017). Próba porównania jakości opieki zdrowotnej w województwach mazowieckim i podlaskim- badania pilotażowe. Pielęgniarstwo Pol..

[2] Szpringer M., Chmielewski J., Kosecka J., Sobczyk B., Komendacka O. (2015). Poziom satysfakcji pacjenta jako jeden z aspektów jakości opieki medycznej. Med. Ogólna Nauk. Zdrowiu.

[3] Cueva-Ariza L., Romero-García M., Delgado-Hito P., Acosta-Mejuto B., Jover-Sancho C., Ricart-Basagaña M.T., Juandó-Prats C., Solà-Solé N., Solà-Ribó M. (2014). Development of an instrument to measure the degree of critical patient’s satisfaction with nursing care: Research protocol. J. Advenced Nurs..

[4] Liao C.C., Li C.R., Lee S.H., Liao W.C., Liao M.Y., Lin J., Yeh C.J., Lee M.C. (2015). Social support and mortality among the aged people with major diseases or ADL disabilities in Taiwan: A national study. Arch. Gerontol. Geriatr..

[5] Zarzycka D. (2000). Podstawy teoretyczne wsparcia społecznego. Probl. Pielęgniarstwa.

[6] Glińska J., Adamska E., Brosowska B., Lewandowska M. (2009). Problemy fizyczne chorych w terminalnej fazie choroby nowotworowej a wsparcie społeczne ze strony personelu pielęgniarskiego. Probl. Pielęgniarstwa.

[7] Sęk H., Cieślak R., Sęk H., Cieślak R. (2011). Wsparcie społeczne—sposoby definiowania, rodzaje i źródła wsparcia, wybrane koncepcje teoretyczne. Wsparcie Społeczne Stres i Zdrowie.

[8] Grochans E., Wieder-Huszla S., Jurczak A., Stanisławska M., Janic E., Szych Z. (2009). Wsparcie emocjonalne jako wyznacznik jakości opieki pielęgniarskiej. Probl. Hig. Epidemiol..

[9] From Loneliness to Social Connection: Charting a Path to Healthier Societies. Report of the WHO Commision on Socjal Connection. https://www.ncbi.nlm.nih.gov/books/NBK585650/.

[10] Kowalczyk M. (2012). Miłość i gniew Koszty emocjonalne rodzin w kontekście opieki nad bliskim chorym. Med. Paliatywna Prakt..

[11] Janowicz A. (2014). Rola opiekunów nieformalnych w opiece u kresu życia. Przyczynek do badań w ramach projektu European Palliative Care Academy (EUPCA). Pielęgniarstwo Zdr. Publiczne.

[12] Marzec A., Walasek L., Andruszkiewicz A., Banaszkiewicz M. (2014). Poczucie koherencji, akceptacja choroby a funkcjonowanie w chorobie przewlekłej osób chorych na chorobę nerek i chorych na cukrzycę. Probl. Pielęgniarstwa.

[13] Rijpkema C., Verweij L., Jepma P., Latour C.H.M., Petersa R.J.G., Reimer W.J.M.S.O., Buurman B.M. (2020). Przebieg ponownej hospitalizacji u słabych starszych pacjentów kardiologicznych. J. Adw. Pielęgniarki.

[14] Schultz B., Corbett C., Hughes R., Bell N. (2021). Przegląd zakresu Wsparcie społeczne wpływa na wskaźniki ponownych przyjęć do szpitala. J. Clin. Nurs..

[15] Müller C., Lautenschläger S., Meyer G., Stephan A. (2017). Interventions to support people with dementia and their caregivers during the transition from home care to nursing home care: A systematic review. Int. J. Nurs. Stud..

[16] Świerżewska D. (2010). Satysfakcja z życia aktywnych i nieaktywnych osób po 60. roku życia. Psychol. Rozw..

[17] Olek D., Uchmanowicz I., Chudiak A., Jankowska-Polańska B. (2014). Wpływ akceptacji choroby na jakość życia chorych w przewlekłej obturacyjnej chorobie płuc. Nurs. Top..

[18] Brzyski P., Knurowski T., Tobiasz-Adamczyk B. (2005). Trafność i rzetelność Skali Wsparcia Społecznego SSL-12-I w populacji osób starszych wiekiem w Polsce. Przegląd Epidemiol..

[19] Kowalczyk-Fobka M. (2013). Wsparcie społeczne w chorobie nowotworowej. Psychoonkologia.

[20] Kurowska K., Bystryk R. (2013). Rola wsparcia i przekonań dotyczących zdrowia w zmaganiu się z problemami wieku geriatrycznego. Geriatria.

[21] Kózka M., Płaszewska-Żywno L. (2010). Model Opieki Pielęgniarskiej nad Chorym Dorosłym. Podręcznik dla Studiów Medycznych.

[22] Izdebski P., Matusik P., Tujakowski J. (2008). Otrzymywane wsparcie społeczne przez partnerów kobiet chorych na raka. Psychoonkologia.

[23] Lorencowicz R., Jasik J., Komar E., Przychodzka E. (2013). Wpływ wsparcia społecznego dla jakości codziennego funkcjonowania osoby chorej na stwardnienie rozsianie. Pielęgniarstwo Neurol. Neurochir..

